# Methanogenesis couples with arsenic methylation in urban interface biofilms under arsenic-phosphorus decoupling stress

**DOI:** 10.3389/fmicb.2026.1798642

**Published:** 2026-03-09

**Authors:** Bangxiao Zheng, Yingsen Lei, Yunwei Lin, Mingxi Zhou, Qingfang Bi

**Affiliations:** 1 Center for Ecology & Health Innovative Research, Xiamen University of Technology, Xiamen, China; 2 Xiamen Key Laboratory of Membrane Research and Application, Xiamen, China; 3 Faculty of Biological and Environmental Sciences, Ecosystems and Environment Research Programme, University of Helsinki, Lahti, Finland; 4 CREAF, Cerdanyola del Vallès, Barcelona, Spain; 5 School of Environmental Science and Engineering, Xiamen University of Technology, Xiamen, China; 6 School of Environment, Nanjing Normal University, Nanjing, China; 7 Department Biogeochemical Integration, Max Planck Institute for Biogeochemistry, Jena, Germany; 8 Institute of Agricultural and Nutritional Sciences, Soil Biogeochemistry, Martin Luther University Halle-Wittenberg, Halle (Saale), Germany; 9 German Centre for Integrative Biodiversity Research (iDiv) Halle-Jena-Leipzig, Leipzig, Germany

**Keywords:** arsenic methylation, arsenic-phosphorus decoupling, functional genes, methanogenesis, urban biofilm

## Abstract

**Introduction:**

Urban interface biofilms represent understudied microenvironments where atmospheric deposition, microbial colonization, and stormwater runoff intersect, yet their role in contaminant transformation and greenhouse gas dynamics remains poorly understood.

**Methods:**

Here, we investigated arsenic-phosphorus biogeochemistry and microbial functional gene dynamics across 18 urban interface sites.

**Results:**

We discovered a striking arsenic-phosphorus decoupling pattern, with industrial sites exhibiting high arsenic (92.1 mg kg^−1^) but low phosphorus (322 mg kg^−1^) concentrations, contrasting with control sites showing the opposite pattern. This decoupling, driven by differential rain-washing dynamics (24.5% As loss vs. 13.5% P loss), created unique selective pressure that drove co-enrichment of arsenic methylation (*arsM*) and high-affinity phosphate transporter (*pstS*) genes (*r* = 0.88, *p* < 0.001). Methylated arsenic species (MMA + DMA) comprising 18%–27% of total arsenic in stormwater runoff provided direct evidence of active *arsM*-mediated biotransformation. Most significantly, we found strong coupling between arsenic methylation and methanogenic potential, with *arsM* showing remarkable correlations with *mcrA* (*r* = 0.99) and *dsrB* (*r* = 0.98). Microcosm incubations confirmed this pattern, revealing CH_4_ production rates positively correlated with *arsM* abundance while CO_2_ flux showed an inverse trend, suggesting arsenic contamination shifts microbial carbon metabolism toward anaerobic pathways. Microbial community analysis revealed selective enrichment of arsenic-tolerant genera (*Acinetobacter*, *Pseudomonas*) and reduced alpha diversity at contaminated sites.

**Discussion:**

These findings establish urban interfaces as previously unrecognized hotspots where arsenic transformation and greenhouse gas production are mechanistically coupled, with important implications for understanding urban biogeochemical cycles and their environmental impacts.

## 1 Introduction

Arsenic (As) contamination represents a significant environmental and public health concern in urban ecosystems, where anthropogenic activities continuously introduce this toxic metalloid through industrial emissions, traffic-derived particles, and construction materials (Huang et al., 2021; Werkenthin et al., 2014). Urban surfaces, including building facades, bridge piers, and concrete walls, function as important interfaces where atmospheric deposition, biofilm colonization, and stormwater runoff intersect to create dynamic biogeochemical microenvironments (Gorbushina, 2007; Gadd, 2017; Zheng et al., 2024). These vertical urban interfaces accumulate pollutants including heavy metals and metalloids, yet their role in contaminant transformation and transport remains poorly understood compared to traditional environmental compartments such as soils and sediments (Zheng and Herr, 2025).

Microbial communities colonizing urban surfaces play critical roles in element cycling and contaminant transformation (Cutler et al., 2013; Li et al., 2019). Among the various biotransformation pathways for arsenic, methylation mediated by the *arsM* gene (encoding arsenic methyltransferase) is of particular significance because it converts inorganic arsenic species into methylated forms such as monomethylarsonic acid (MMA) and dimethylarsinic acid (DMA), thereby altering arsenic mobility, bioavailability, and toxicity (Qin et al., 2006; Zhu et al., 2014). The *arsM* gene is widely distributed among bacteria and archaea, including taxa commonly found in soil and aquatic biofilms (Jia et al., 2013).

Classical biogeochemistry recognizes arsenic and phosphorus as chemical analogs due to their similar atomic structures and competitive interactions for sorption sites and biological uptake pathways (Rosen et al., 2011; Elias et al., 2012). In soil and aquatic systems, arsenate [As(V)] and phosphate share common transport mechanisms, leading to the expectation that their distributions should be positively correlated (Zhao et al., 2010). However, urban interfaces present unique conditions where anthropogenic As inputs may occur independently of phosphorus dynamics, potentially disrupting this expected relationship. The consequences of As-P decoupling for microbial functional gene expression and arsenic transformation pathways have not been previously explored.

From an environmental perspective, the potential linkage between arsenic transformation and greenhouse gas (GHG) production merits investigation. Many microorganisms capable of arsenic methylation, including certain *Methanosarcina* species and sulfate-reducing bacteria, are also involved in anaerobic carbon metabolism pathways that generate methane (CH_4_) or are coupled to methanogenesis (Qin et al., 2006; Wang et al., 2015). Recent studies on paddy soils and constructed wetlands have further demonstrated that arsenic mobilization and greenhouse gas emissions are mechanistically interlinked, and that amendment strategies such as nitrate, birnessite, and zero-valent iron/manganese can simultaneously suppress arsenic release and reduce GHG emissions (Chen Y. et al., 2025; Chen et al., 2025a; Zhao et al., 2025). The gene encoding methyl-coenzyme M reductase serves as a functional marker for methanogenic archaea, while *dsrB* encoding dissimilatory sulfite reductase indicates sulfate-reducing bacteria capable of anaerobic metabolism (Steinberg and Regan, 2008; Wagner et al., 1998). Whether urban interface biofilms harbor conditions favorable for both arsenic methylation and anaerobic carbon metabolism remains unknown, yet such coupling would have important implications for understanding urban biogeochemical cycles and their environmental impacts.

Here, we investigate arsenic transformation potential and its relationship to phosphorus dynamics and GHG-related gene enrichment in urban interface biofilms across a contamination gradient in Xiamen, China. We hypothesize that: (1) anthropogenic arsenic contamination creates As-P decoupling on urban surfaces, contrasting with the typical positive As-P relationship observed in natural systems; (2) dual stress from high As and low bioavailable P drives co-selection of arsenic transformation genes (*arsM*) and high-affinity phosphate uptake genes (*pstS*); (3) methylated arsenic species in stormwater runoff provide functional evidence of active microbial transformation; and (4) anaerobic metabolism genes (*mcrA*, *dsrB*) are co-enriched with *arsM*, revealing potential coupling between arsenic methylation and GHG production in urban biofilms. Our findings reveal urban interfaces as previously unrecognized hotspots for coupled arsenic transformation and anaerobic carbon metabolism, with implications for urban contaminant cycling and environmental impact assessment.

## 2 Materials and methods

### 2.1 Study area, sampling design and sample collection

This study was conducted in Xiamen, a subtropical coastal city in southeastern China (24°26′–24°54′N, 117°53′–118°26′E). The sampling campaign spanned from May 2024 to December 2024, covering one complete wet–dry seasonal cycle. We established 18 sampling sites across six urban interface types representing a gradient of arsenic exposure: Industrial-High (IH, *n* = 3), Industrial-Low (IL, *n* = 3), Traffic-High (TH, *n* = 3), Traffic-Low (TL, *n* = 3), Control-Campus (CC, *n* = 3), and Control-Gulangyu (CG, *n* = 3). All samples were collected from vertical surfaces at 1.3 m height, where biofilm development is representative of urban interface conditions (Gorbushina, 2007). Detailed site descriptions are provided in Supplementary Table S1.

Surface biofilm samples were collected during the wet season (May–July 2024) and subsequent dry season (October–December 2024) using sterile brushes and spatulas (Cutler et al., 2013). At each site, approximately 5–10 g of material was scraped from a 30 × 30 cm quadrat. In addition to seasonal sampling, paired rain-event sampling was conducted during the wet season at the same 18 sites. For each of four qualifying rain events (cumulative rainfall >15 mm), biofilm was collected within 24 h before and 24–48 h after the event from adjacent, non-overlapping quadrats (separated by ~10 cm) to avoid depletion bias; centimeter-scale spatial heterogeneity may introduce minor variability but is preferable to systematic underestimation from re-scraping a disturbed surface. Stormwater runoff (500 mL) was collected from the base of sampled interfaces during each event. All samples were transported on ice and stored at −80 °C until analysis.

### 2.2 Chemical and molecular analyses

Total As was determined by ICP-MS (Agilent 7,700x) after HNO₃–HF-HClO₄ digestion (Huang et al., 2018). Total P was measured colorimetrically (Murphy and Riley, 1962), and bioavailable P was extracted with NaHCO₃ (Olsen et al., 1954). Arsenic speciation in runoff [As(III), As(V), MMA, DMA] was determined by HPLC-ICP-MS (Gong et al., 2020). Phosphorus fractions (SRP, IP, OP) were analyzed following Worsfold et al. (2016). Quality control procedures and auxiliary parameters (pH, Fe/Al oxides, TOC) are detailed in Supplementary Tables S2, S3.

Loss rate was calculated as: Loss (%) = (C_before_ − C_after_) / C_before_ × 100%, where C_before_ and C_after_ represent total element concentrations (mg kg^−1^ dry weight) in biofilm samples collected from adjacent quadrats before and after each rain event, respectively. Reported values are means across four rain events per site.

DNA was extracted using the DNeasy PowerSoil Pro Kit (Qiagen). The 16S rRNA gene V4 region was amplified with primers 515F/806R (Caporaso et al., 2011) and sequenced on Illumina NovaSeq 6000. Sequences were processed in QIIME2 with DADA2 denoising (Bolyen et al., 2019; Callahan et al., 2016) and assigned taxonomy against SILVA v138 (Quast et al., 2013).

Functional gene abundances were quantified by qPCR on a QuantStudio 6 Flex system. Target genes included arsenic transformation genes (*arsC*, *arsM*), phosphorus acquisition genes (*pstS*, *phoD*), and anaerobic metabolism markers (*mcrA*, *dsrB*), following the functional gene quantification framework described by Zheng et al. (2018). Primer sequences, thermal conditions, and standard curve parameters are provided in Supplementary Table S4.

### 2.3 Greenhouse gas production potential

Microcosm incubations were conducted following Angel et al. (2012). Fresh biofilm (5 g wet weight) was mixed with 10 mL sterile deionized water to standardize moisture content and transferred to 120-mL serum bottles. Headspace was flushed with N_2_ (99.999%) for 5 min and bottles were sealed with butyl rubber stoppers and aluminum crimps to establish anaerobic conditions. Incubations were conducted at 25 °C ± 1 °C in the dark for 14 days. Headspace CH_4_ and CO_2_ concentrations were measured at days 1, 3, 7, and 14 by gas chromatography (Shimadzu GC-2014, equipped with FID and TCD detectors). Gas fluxes were calculated from the linear increase in headspace concentration over the incubation period, normalized to the surface area of the original sampling quadrat (0.09 m^2^) from which the biofilm was collected. Detailed incubation protocols are provided in Supplementary Section S4.

### 2.4 Statistical analyses

Statistical analyses were performed in R v4.3.1 (R Core Team, 2023). Correlations were assessed using Pearson’s coefficient. Principal component analysis was conducted with the *vegan* package (Oksanen et al., 2022). Group differences were tested by one-way ANOVA with Tukey’s HSD, and rain-event effects by paired *t*-tests (*α* = 0.05).

## 3 Results

### 3.1 Arsenic-phosphorus decoupling across urban Interface types

We define As-P decoupling as the inverse spatial relationship between total As and total P concentrations across urban interface types (i.e., a significant negative correlation), which contrasts with the positive As-P correlation expected from shared geochemical behavior in natural systems. This decoupling is further reinforced dynamically by differential rain-washing loss rates (Section 3.4) and is reflected in discordant bioavailable fractions across the gradient.

**Figure 1:**
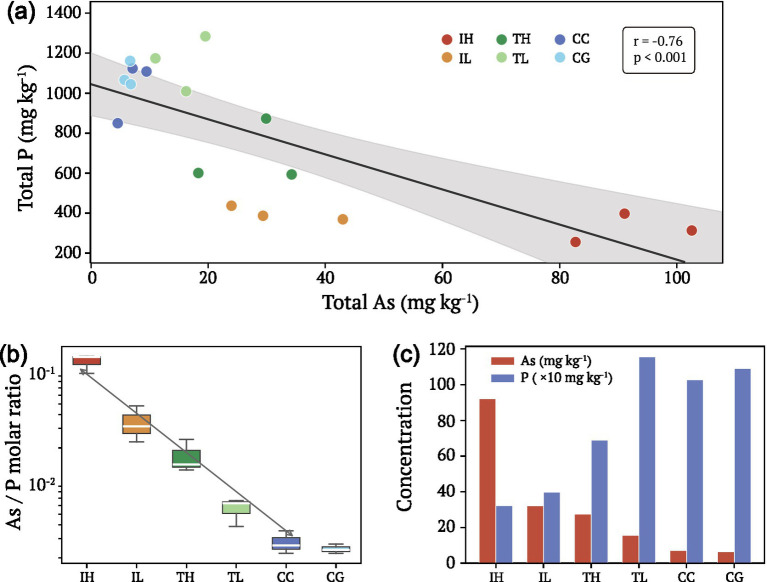
Arsenic-phosphorus decoupling across urban interface types in Xiamen, China. **(a)** Scatter plot showing the negative correlation between total arsenic (As) and total phosphorus (P) concentrations across 18 urban interface samples. The solid line represents linear regression (*r* = −0.76, *p* < 0.001), with the shaded area indicating 95% confidence interval. **(b)** Box plots of As/P molar ratio across interface groups displayed on a logarithmic scale, demonstrating approximately 50-fold gradient from industrial-high to control sites. **(c)** Comparison of mean As (red bars) and P (blue bars, scaled ×10) concentrations across groups, illustrating inverse distribution patterns. Interface groups: IH, Industrial-High; IL, Industrial-Low; TH, Traffic-High; TL, Traffic-Low; CC, Control-Campus; CG, Control-Gulangyu. Statistical analysis of correlations is presented in Supplementary Table S3.

Total As and P concentrations exhibited a striking negative correlation across the 18 urban interface samples (*r* = −0.76, *p* < 0.001; Figure 1a; Supplementary Table S3). IH sites contained the highest As concentrations (92.1 ± 10.0 mg kg^−1^) but the lowest P levels (322 ± 71 mg kg^−1^), whereas control sites showed the opposite pattern (CC: As = 7.0 ± 2.5 mg kg^−1^, *p* = 1,027 ± 154 mg kg^−1^; Table 1). The As/P molar ratio varied by approximately 50-fold across the pollution gradient, from 0.121 at IH sites to 0.002 at CG sites (Figure 1b). This inverse relationship was further illustrated by the opposing concentration patterns of As and P across interface types (Figure 1c), establishing a clear As-P decoupling phenomenon on urban surfaces.

**Table 1:** Physicochemical and microbial characteristics across urban interface types.

Interface type	As (mg/kg)	P (mg/kg)	As/P	*arsC*	*arsM*	*pstS*
Industrial-High	92.1 ± 10.0	322 ± 71	0.121 ± 0.023	7.34 ± 0.25	6.90 ± 0.15	7.13 ± 0.24
Industrial-Low	32.1 ± 9.8	398 ± 35	0.034 ± 0.013	6.83 ± 0.43	6.42 ± 0.34	6.92 ± 0.21
Traffic-High	27.5 ± 8.2	689 ± 159	0.017 ± 0.006	6.41 ± 0.13	6.01 ± 0.18	6.65 ± 0.02
Traffic-Low	15.6 ± 4.3	1,156 ± 138	0.006 ± 0.002	6.05 ± 0.13	5.60 ± 0.16	6.49 ± 0.13
Control-Campus	7.0 ± 2.5	1,027 ± 154	0.003 ± 0.001	5.64 ± 0.19	5.28 ± 0.34	6.14 ± 0.22
Control-Gulangyu	6.4 ± 0.6	1,091 ± 62	0.002 ± 0.0002	5.76 ± 0.11	5.55 ± 0.14	6.12 ± 0.08

Values are mean ± SD. Functional gene abundances are expressed as log_10_ copies g^−1^ dry weight.

### 3.2 Functional gene enrichment and co-selection patterns

**Figure 2:**
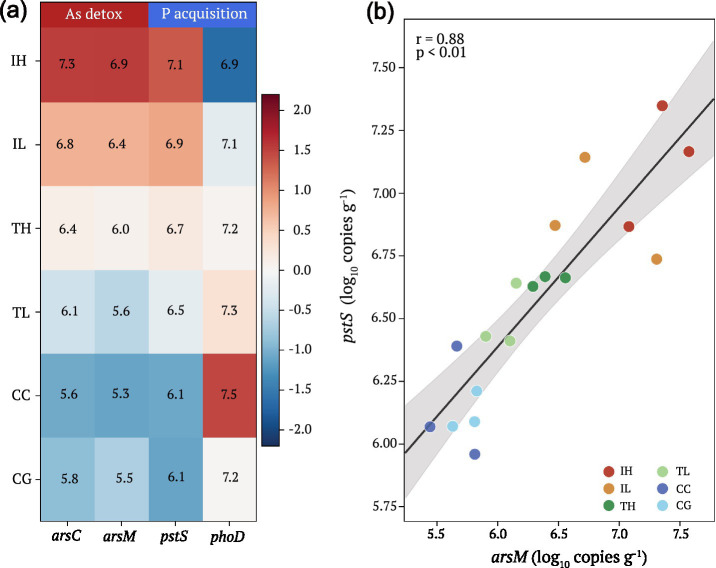
Functional gene enrichment patterns and co-selection of arsenic transformation and phosphorus acquisition genes. **(a)** Heatmap of functional gene abundances (*Z*-score normalized) across interface groups. The top color bar indicates gene function: As detoxification genes (*arsC*, *arsM*; red) and P acquisition genes (*pstS*, *phoD*; blue). Values within cells represent log_10_ copies g^−1^ dry weight. **(b)** Positive correlation between *arsM* (arsenic methyltransferase) and *pstS* (high-affinity phosphate transporter) gene abundances (*r* = 0.80, *p* < 0.001), demonstrating co-selection under dual As-P stress conditions. The annotation highlights the “co-selection under high As/low P stress” concept.

Arsenic transformation genes (*arsC*, *arsM*) and the phosphate transporter gene (*pstS*) showed parallel enrichment patterns across interface types, with highest abundances at IH sites and lowest at control sites (Figure 2a; Table 1). In contrast, the alkaline phosphatase gene (*phoD*) displayed an inverse pattern, with elevated abundances at P-rich control sites (Table 1). Notably, *arsM* and *pstS* abundances were strongly positively correlated (*r* = 0.80, *p* < 0.001; Figure 2b), indicating co-selection of arsenic methylation capacity and high-affinity phosphate uptake under dual As-P stress. The correlation matrix (Supplementary Table S3; Supplementary Figure S1) revealed strong positive associations among As-related variables (As, *arsC*, *arsM*, *pstS*; r > 0.76) and their collective negative associations with P-related variables (P, OlsenP, *phoD*).

### 3.3 Arsenic speciation and methylation in stormwater runoff

**Figure 3:**
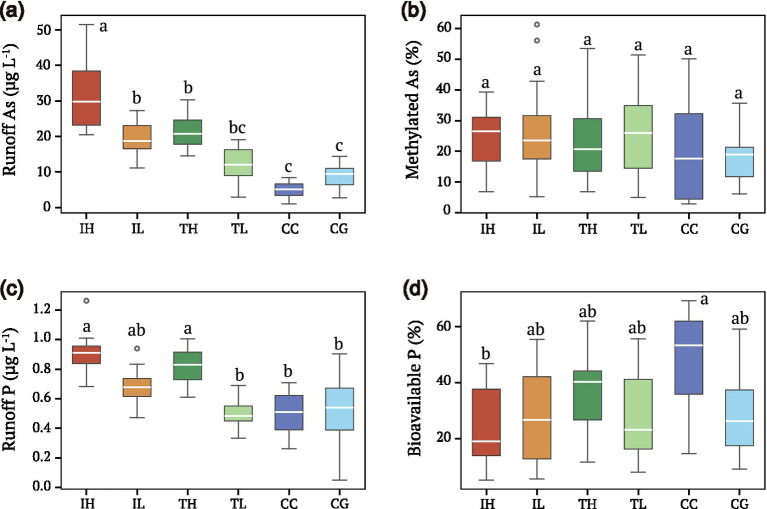
Arsenic speciation in stormwater runoff reveals microbial methylation activity. **(a)** Box plots of total As concentration in runoff across interface groups (μg L^−1^). **(b)** Methylated As percentage (MMA + DMA) in runoff, indicating microbial arsenic transformation activity. Higher values suggest more active microbial processing via *arsM*-mediated methylation. **(c)** Total P concentration in runoff (mg L^−1^). **(d)** Bioavailable P (soluble reactive phosphorus, SRP) percentage in runoff. Data represent four rain events (*n* = 12 per group). Different lowercase letters indicate significant differences among groups (one-way ANOVA with Tukey’s HSD, *p* < 0.05). Detailed speciation breakdown is shown in Supplementary Figure S4.

Runoff samples revealed substantial microbial transformation of arsenic (Figure 3; Table 2). Total As in runoff ranged from 5.0 ± 2.2 μg L^−1^ at CC sites to 32.0 ± 10.1 μg L^−1^ at IH sites (Figure 3a), reflecting the source concentrations on interface surfaces. Critically, methylated arsenic species (MMA + DMA) comprised 18–27% of total runoff As across all groups (Figure 3b; Table 2), providing direct evidence of *arsM*-mediated biotransformation. Runoff P concentrations were highest at industrial sites (IH: 0.86 ± 0.18 mg L^−1^; Figure 3c), while bioavailable P (SRP) percentage was greatest at control sites (CC: 48%; Figure 3d). Detailed speciation breakdowns (Supplementary Figure S4) showed that As(V) dominated inorganic As fractions at most sites, while SRP comprised the largest P fraction at control sites.

**Table 2:** Arsenic speciation and methylation potential in stormwater runoff.

Interface type	Total As (μg/L)	MMA (%)	DMA (%)	Methylated As (%)	Total P (mg/L)
Industrial-High	32.0 ± 10.1	9.1 ± 5.9	14.7 ± 11.7	23.8 ± 11.3	0.86 ± 0.18
Industrial-Low	19.7 ± 4.9	6.4 ± 8.0	20.9 ± 18.3	27.2 ± 18.0	0.69 ± 0.14
Traffic-High	21.5 ± 4.9	10.6 ± 7.4	12.6 ± 10.2	23.2 ± 13.3	0.83 ± 0.12
Traffic-Low	12.1 ± 4.9	14.1 ± 13.9	11.5 ± 10.0	25.6 ± 15.3	0.48 ± 0.10
Control-Campus	5.0 ± 2.2	13.6 ± 16.1	7.0 ± 7.3	20.7 ± 17.2	0.48 ± 0.10
Control-Gulangyu	8.8 ± 3.5	8.5 ± 8.1	9.8 ± 7.4	18.3 ± 9.0	0.54 ± 0.17

Values are mean ± SD from 4 rain events (*n* = 12 per group). MMA, monomethylarsonic acid; DMA, dimethylarsinic acid; Methylated As, MMA + DMA.

### 3.4 Differential rain-washing dynamics

The four rain events used for paired sampling had cumulative rainfall of 18.5, 22.3, 31.0, and 45.2 mm, with durations of 4–12 h and peak intensities ranging from 5.2 to 18.6 mm h^−1^. Although event magnitude varied, the direction of differential As vs. P loss was consistent across all four events, with As loss exceeding P loss in every event at every site category.

**Figure 4:**
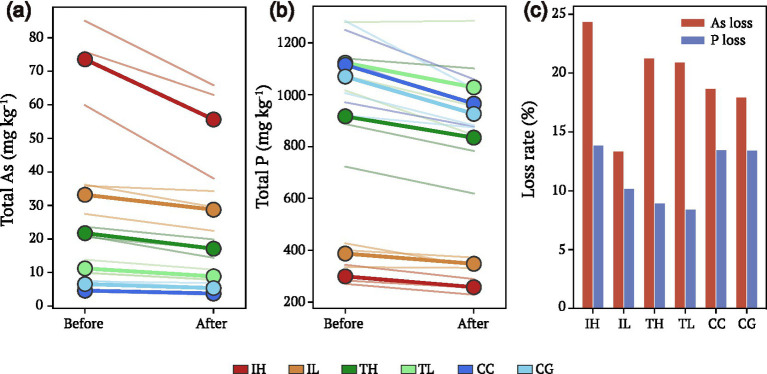
Differential rain-washing dynamics of arsenic and phosphorus on urban interfaces. **(a)** Slope chart showing As concentration changes before and after a rain event. Thin lines represent individual samples; thick lines with solid markers indicate group means. **(b)** Slope chart showing P concentration changes, demonstrating proportionally smaller loss compared to As. **(c)** Comparison of As (red bars) and P (blue bars) loss rates (%) across groups. Arsenic shows consistently higher loss rates than phosphorus, especially at industrial sites (IH: 24.5% vs. 13.5%), contributing to progressive As-P decoupling over time.

Paired before/after rain-event sampling revealed differential loss dynamics for As and P (Figure 4). Both elements decreased following rain events, but As showed consistently higher proportional losses than P across all interface types (Figures 4a,b). At IH sites, mean As loss reached 24.5% compared to only 13.5% P loss (Figure 4c). This differential washout was most pronounced at high-exposure industrial and traffic sites, where As is presumably present in more water-soluble or loosely-bound forms. The greater mobility of As relative to P during rain events provides a dynamic mechanism that reinforces the As-P decoupling pattern observed in the static concentration data (Figure 1).

### 3.5 Multivariate ordination of chemical-microbial relationships

**Figure 5:**
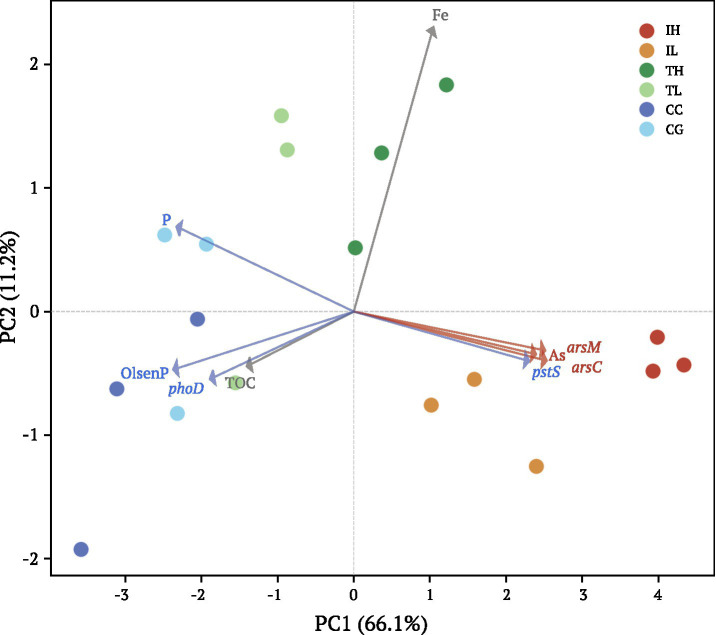
Principal component analysis of chemical and microbial parameters across urban interfaces. PCA biplot showing sample distribution and variable loadings. PC1 (66.1%) and PC2 (11.2%) together explain 77.3% of total variance. Red arrows indicate As-related variables (As, *arsC*, *arsM*, *pstS*); blue arrows indicate P-related variables (P, OlsenP, *phoD*); gray arrows represent other parameters (Fe, TOC). The opposing directions of As-related and P-related vectors along PC1 visually confirm the As-P decoupling pattern. Industrial sites (IH, IL) cluster in the positive PC1 region associated with high As and functional gene abundances, while control sites (CC, CG) occupy the negative PC1 region characterized by high P availability. Correlation coefficients supporting this ordination are provided in Supplementary Table S3.

PCA captured 77.3% of total variance in the first two axes (Figure 5; Supplementary Figure S1). PC1 (66.1%) clearly separated samples along the As-P gradient, with IH and IL sites clustering in the positive region associated with high As concentrations and elevated *arsC*, *arsM*, and *pstS* abundances, while CC and CG sites occupied the negative region characterized by high P availability and *phoD* enrichment. The opposing orientations of As-related and P-related variable loadings along PC1 visually confirmed the decoupling pattern. Traffic sites (TH, TL) occupied intermediate positions, consistent with their moderate exposure levels.

### 3.6 Seasonal variation in functional gene abundances

**Figure 6:**
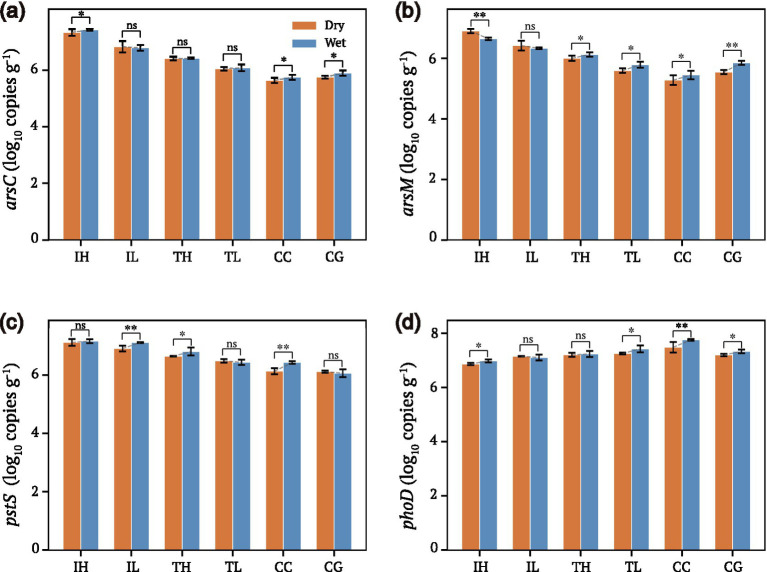
Seasonal variation in arsenic transformation and methanogenesis gene abundances. **(a)** *arsC* (arsenate reductase) abundances in dry (orange) versus wet (steel blue) seasons. **(b)** *arsM* (arsenic methyltransferase) abundances showing elevated levels in wet season, particularly at high-exposure sites. **(c)** *pstS* (phosphate transporter) abundances with relatively stable patterns across seasons. **(d)** *phoD* (alkaline phosphatase) abundances across seasons. Top color bars indicate gene function category: As-related (red) or P-related (blue). Error bars represent standard error of the mean (*n* = 3 per group). Asterisks denote significant seasonal differences within each group (paired *t*-test; ^*^p < 0.05, ^**^p < 0.01, ns = not significant; *n* = 3). Seasonal data are detailed in Supplementary Table S2.

Arsenic transformation genes showed modest seasonal variation, with *arsC* and *arsM* abundances slightly elevated during the wet season, particularly at high-exposure sites (Figures 6a,b; Supplementary Table S2). The *pstS* gene exhibited relatively stable abundances across seasons (Figure 6c), suggesting constitutive expression of high-affinity phosphate uptake under chronic P limitation. The *phoD* gene also showed minimal seasonal variation (Figure 6d). The wet season enhancement of *arsM* is consistent with the elevated methylated As fractions observed in wet-season runoff (Figure 3b), linking gene abundance dynamics to functional outputs.

### 3.7 Coupling of arsenic methylation and methanogenesis genes

Methanogenesis marker gene *mcrA* and sulfate reduction marker *dsrB* abundances followed remarkably similar spatial patterns to arsenic transformation genes (Table 3). Both *mcrA* (*r* = 0.99, *p* < 0.001) and *dsrB* (*r* = 0.98, *p* < 0.001) were strongly positively correlated with *arsM*, indicating co-enrichment of anaerobic carbon metabolism potential at high-As interfaces. The high correlations between *arsM* and *mcrA*/*dsrB* (*r* = 0.99 and 0.98) are computed across all 18 samples spanning six interface groups and are partly driven by between-group variance along the contamination gradient; within-group assessment was precluded by limited replication (*n* = 3). Leave-one-out analysis confirmed that no single sample reduced these correlations below *r* = 0.95. Although no formal multiple-testing correction was applied to the full correlation matrix, all key correlations (*arsM*–*mcrA*, *arsM*–*dsrB*, *arsM*–*pstS*) remain significant under Bonferroni adjustment (*k* = 55 pairwise tests among 11 variables; adjusted *α* = 0.00091; all reported *p* < 0.001). We therefore interpret these values as reflecting gradient-level co-enrichment rather than evidence of direct mechanistic coupling at the individual-sample scale.

**Table 3:** Anaerobic carbon metabolism potential and greenhouse gas-related gene abundances across urban interface types.

Interface type	*mcrA*	*dsrB*	*arsM*	CH₄ flux	CO₂ flux	TOC (g kg^−1^)
Industrial-High	6.25 ± 0.18	4.95 ± 0.15	6.90 ± 0.15	38.4 ± 11.6	152.0 ± 18.7	27.2 ± 5.9
Industrial-Low	5.95 ± 0.22	4.70 ± 0.21	6.42 ± 0.34	27.9 ± 9.4	163.5 ± 21.3	28.7 ± 8.0
Traffic-High	5.75 ± 0.20	4.55 ± 0.18	6.01 ± 0.18	21.6 ± 8.8	210.8 ± 24.9	34.1 ± 6.2
Traffic-Low	5.55 ± 0.17	4.45 ± 0.16	5.60 ± 0.16	14.8 ± 6.7	222.4 ± 26.5	35.5 ± 7.1
Control-Campus	5.40 ± 0.25	4.30 ± 0.24	5.28 ± 0.34	11.7 ± 5.9	201.3 ± 22.1	33.8 ± 4.9
Control-Gulangyu	5.60 ± 0.16	4.50 ± 0.14	5.55 ± 0.14	18.2 ± 7.9	189.6 ± 19.4	31.2 ± 3.8

Values are mean ± SD (*n* = 3). mcrA, methyl-coenzyme M reductase (methanogenesis marker); dsrB, dissimilatory sulfite reductase (sulfate reduction marker); arsM, arsenite S-adenosylmethionine methyltransferase (arsenic methylation marker); TOC, total organic carbon. Gene abundances expressed as log_10_ copies g^−1^ dry weight. CH_4_ fluxes expressed as μg C m^−2^ h^−1^; CO_2_ fluxes expressed as mg C m^−2^ h^−1^.

Microcosm incubations confirmed this pattern: CH_4_ production rates were highest at IH sites (38.4 ± 11.6 μg C m^−2^ h^−1^) and lowest at CC sites (11.7 ± 5.9 μg C m^−2^ h^−1^), strongly correlating with *arsM* abundance (*r* = 0.98; Table 3). Interestingly, CO_2_ flux showed an inverse pattern, with highest values at traffic and control sites (TL: 222.4 mg C m^−2^ h^−1^) and lowest at IH sites (152 mg C m^−2^ h^−1^), suggesting that high As levels may shift microbial carbon metabolism from aerobic respiration toward anaerobic pathways including methanogenesis.

### 3.8 Microbial community responses to as-P stress

Alpha diversity metrics revealed reduced community diversity under high As stress (Supplementary Figure S2). Shannon diversity was lowest at IH sites (3.00 ± 0.02) and highest at control sites (CC: 3.15 ± 0.00; CG: 3.15 ± 0.01), with a significant negative correlation between Shannon index and As concentration (*r* = −0.84, *p* < 0.001; Supplementary Table S3). Observed ASVs showed a similar pattern (Supplementary Figure S2b). Community composition analysis (Supplementary Figure S3) revealed distinct taxonomic signatures across interface types. *Acinetobacter* and *Pseudomonas*, genera known for arsenic resistance and metabolic versatility, were enriched at IH sites, while *Sphingomonas* and *Methylobacterium* dominated at control sites. These community shifts align with the functional gene patterns and support selective enrichment of As-tolerant taxa at contaminated interfaces.

## 4 Discussion

This study provides the first comprehensive characterization of arsenic-phosphorus biogeochemistry and microbial functional gene dynamics in urban interface biofilms. Our results reveal a striking As-P decoupling pattern across the contamination gradient, with industrial sites exhibiting high arsenic but low phosphorus concentrations, contrary to the positive correlation typically observed in natural systems where arsenate and phosphate share similar geochemical behavior (Zhao et al., 2010; Rosen et al., 2011). This decoupling reflects the distinct anthropogenic sources of arsenic in urban environments, primarily atmospheric deposition from industrial processes and traffic emissions, which operate independently of phosphorus inputs derived mainly from biological activity and construction materials (Huang et al., 2021; Werkenthin et al., 2014). The approximately 50-fold gradient in As/P molar ratios across interface types demonstrates that urban surfaces create unique biogeochemical niches distinct from soil or aquatic systems, where such extreme decoupling is rarely encountered.

The differential rain-washing dynamics we observed provide a mechanistic explanation for the progressive As-P decoupling on urban interfaces. The consistently higher proportional loss of arsenic compared to phosphorus during rain events (24.5% vs. 13.5% at industrial sites) suggests that arsenic exists in more water-soluble or loosely-bound forms, possibly associated with atmospheric particulates deposited on surfaces (Cutler et al., 2013). In contrast, phosphorus appears more strongly retained, likely through biological immobilization in biofilms or stronger binding to surface materials. This differential mobility has important implications for understanding contaminant transport from urban surfaces to stormwater systems and receiving waters (Gong et al., 2020).

Our central finding is the co-selection of arsenic methylation capacity (*arsM*) and high-affinity phosphate uptake (*pstS*) under dual As-P stress. The strong positive correlation between these genes (*r* = 0.80) indicates that microorganisms in high-As, low-P environments simultaneously enhance both arsenic detoxification and phosphorus acquisition capabilities. This co-selection likely reflects the shared regulatory responses to arsenic and phosphorus stress in bacteria, as the *pst* operon is induced under phosphate limitation while also being involved in arsenate uptake due to the chemical similarity between arsenate and phosphate (Elias et al., 2012). Elevated *arsM* expression under these conditions represents an adaptive strategy for arsenic detoxification through methylation, converting the more toxic inorganic forms to less toxic organic species (Qin et al., 2006; Zhu et al., 2014).

The enrichment of *pstS* under low-P conditions parallels observations of inorganic phosphate solubilizing communities responding to phosphorus gradients in soil systems (Zheng et al., 2019a). It is important to note that elevated *pstS* abundance under low-P conditions may also inadvertently increase arsenate [As(V)] uptake, as the high-affinity phosphate transport system (Pst) does not perfectly discriminate between phosphate and arsenate (Elias et al., 2012). This creates a feedback loop in which phosphorus scarcity drives upregulation of Pst transporters, which in turn increases intracellular arsenate exposure and may further select for arsenic detoxification mechanisms including *arsM*-mediated methylation.

The presence of methylated arsenic species (MMA + DMA) comprising 18%–27% of total arsenic in stormwater runoff provides direct functional evidence that the *arsM* genes detected in urban biofilms are actively expressed and mediating arsenic biotransformation. This methylation percentage is comparable to or higher than values reported for agricultural soils (10%–20%) and paddy sediments (15%–25%), highlighting urban interfaces as unexpectedly active sites for arsenic transformation (Jia et al., 2013). The ecological significance of this transformation extends beyond detoxification: methylated arsenic species exhibit different mobility, volatility, and bioavailability compared to inorganic forms, potentially altering the environmental fate and biological impacts of arsenic transported from urban surfaces (Wang et al., 2015).

Perhaps the most significant finding of this study is the strong coupling between arsenic methylation and methanogenic genes across the contamination gradient. The remarkably high correlations of *arsM* with both *mcrA* (*r* = 0.99) and *dsrB* (*r* = 0.98) indicate that arsenic transformation and anaerobic carbon metabolism are co-enriched at high-As interfaces. The co-variation patterns among functional genes observed here are consistent with the network-based framework for interpreting coupled elemental cycling genes (Zheng et al., 2019b). This pattern was confirmed by microcosm incubations showing CH_4_ production rates strongly correlated with *arsM* abundance, while CO_2_ flux exhibited an inverse trend. These results suggest that arsenic contamination may shift microbial carbon metabolism from aerobic respiration toward anaerobic pathways, potentially through the formation of anoxic microniches within biofilms where organic matter accumulation and oxygen diffusion limitation create favorable conditions for both methylotrophic methanogenesis and arsenic methylation (Angel et al., 2012). The co-occurrence of *arsM* and *mcrA* is consistent with the known distribution of arsenic methylation capacity among methanogenic archaea and methylotrophic bacteria, many of which carry both functional capacities (Qin et al., 2006).

The microbial community responses to As-P stress revealed selective enrichment of As-tolerant taxa at contaminated interfaces. The dominance of *Acinetobacter* and *Pseudomonas* at industrial sites is consistent with the well-documented arsenic resistance and metabolic versatility of these genera, which commonly harbor *ars* operons and can thrive under metal stress (Li et al., 2019; Cai et al., 2009). The reduced alpha diversity at high-As sites further indicates that arsenic contamination exerts strong selective pressure, filtering the community toward stress-tolerant specialists at the expense of overall diversity. This pattern mirrors observations from contaminated soils and mining-impacted environments, suggesting that urban interfaces follow similar ecological principles despite their distinct physical and chemical characteristics (Quast et al., 2013; Gadd, 2017).

We note that the observed co-variation between *arsM* and *mcrA*/*dsrB* does not necessarily imply direct mechanistic coupling. Several shared environmental drivers could produce parallel enrichment patterns, including: (i) moisture and oxygen microgradients within biofilm matrices that simultaneously favor both methylotrophic and methanogenic metabolisms; (ii) organic carbon availability, which fuels both anaerobic carbon pathways and provides methyl donors for arsenic methylation; and (iii) the physicochemical characteristics of the urban interface (e.g., surface roughness, mineral composition) that co-select for anaerobic microniche formation. Disentangling these shared drivers from direct metabolic coupling will require metatranscriptomic and stable isotope probing approaches.

Our findings have important implications for understanding urban biogeochemical cycles and their environmental impacts. First, urban interfaces emerge as overlooked compartments for arsenic transformation that can significantly modify the speciation and mobility of arsenic entering stormwater systems. Second, the coupling of arsenic methylation and methanogenic potential suggests that high-As urban surfaces may contribute to both modified arsenic cycling and enhanced methane emissions, a pattern increasingly recognized in other arsenic-affected systems such as flooded paddy soils and constructed wetlands where microbial-mediated arsenic transformation and GHG dynamics are intrinsically linked (Chen Y. et al., 2025; Chen et al., 2025a,b; Zhao et al., 2025), though the absolute magnitude of these fluxes requires further quantification at larger spatial scales. Third, the As-P decoupling stress on microbial communities represents a novel selective environment that shapes functional gene distributions and may select for unique metabolic capabilities not commonly observed in natural systems (Gorbushina, 2007; Cutler et al., 2013).

## 5 Conclusion

This study reveals urban interface biofilms as previously unrecognized hotspots for coupled arsenic transformation and anaerobic carbon metabolism. We demonstrate that anthropogenic arsenic contamination creates pronounced As-P decoupling on urban surfaces, driving co-selection of arsenic methylation (*arsM*) and phosphate acquisition (*pstS*) genes. The presence of methylated arsenic (18%–27%) in stormwater runoff provides direct evidence of active *arsM*-mediated biotransformation. Most significantly, we found strong spatial co-variation between arsenic methylation and methanogenic genes (*mcrA*), with CH_4_ production potential correlating with *arsM* abundance (*r* = 0.98). These findings establish urban interfaces as dynamic biogeochemical reactors where contaminant transformation and greenhouse gas production are functionally co-enriched, expanding our understanding of arsenic cycling in anthropogenic environments.

Several limitations should be acknowledged. First, our study was conducted in a single city with subtropical climate, and the generalizability of these patterns to other urban environments and climate zones requires validation. Second, while we detected strong correlations between functional genes and measured CH_4_ production in microcosms, field-scale gas flux measurements would strengthen the linkage between gene abundance and actual GHG emissions. Third, the mechanisms underlying the *arsM-mcrA* coupling warrant further investigation through metatranscriptomic approaches to confirm active gene expression and identify the specific microbial populations responsible. Future studies should also explore the potential for urban interface management strategies, such as biofilm engineering or surface treatments, to mitigate arsenic transformation and associated environmental impacts.

## Data Availability

The raw 16S rRNA gene sequencing data have been deposited in the NCBI Sequence Read Archive (SRA) under BioProject accession number PRJNA1398822. All other data supporting the findings of this study, including chemical analyses and functional gene quantification results, are available from the corresponding author upon reasonable request.
