# Supplementary material for: Methanogenesis couples with arsenic methylation in urban interface biofilms under arsenic-phosphorus decoupling stress
Source: Front Microbiol. 2026 Mar 9;17:1798642. doi: 10.3389/fmicb.2026.1798642 (PMC13006510; doi:10.3389/fmicb.2026.1798642)
Supplement: Supplementary file 1 [file Presentation_1.pdf]

Supplementary Information for

Methanogenesis couples with arsenic methylation in urban interface biofilms under arsenic-phosphorus decoupling stress

Bangxiao Zheng[1,2,3,4*#], Yingsen Lei[1,5#], Yunwei Lin[5#], Mingxi Zhou[6], Qingfang Bi[7,8,9**]

[1] *Center for Ecology & Health Innovative Research, Xiamen University of Technology, Xiamen 361024, P. R. China;*
[2] *Xiamen Key Laboratory of Membrane Research and Application, Xiamen 361024, P. R. China;*
[3] *Faculty of Biological and Environmental Sciences, Ecosystems and Environment Research Programme, Niemenkatu 73, FI-15140, Lahti, University of Helsinki, Finland;*
[4] *CREAF, Cerdanyola del Vallès, 08193 Barcelona, Catalonia, Spain;*
[5] *School of Environmental Science and Engineering, Xiamen University of Technology, Xiamen 361024, P. R. China;*
[6] *School of Environment, Nanjing Normal University, Beijing 210023, P. R. China;*
[7] *Department Biogeochemical Integration, Max Planck Institute for Biogeochemistry, Jena 07745, Germany;*
[8] *Institute of Agricultural and Nutritional Sciences, Soil Biogeochemistry, Martin Luther University Halle-Wittenberg, 06120, Halle (Saale), Germany;*
[9] *German Centre for Integrative Biodiversity Research (iDiv) Halle-Jena-Leipzig, 04103 Leipzig, Germany.*

[*] Corresponding Author

Phone: +86 137 7465 9187; E-mail: bangxiao.zheng@xmut.edu.cn

[**] Corresponding Author

Phone: +49 3641 57-6249; E-mail: qbi@bgc-jena.mpg.de

# These authors contributed to this study equally.

Supplementary materials and methods

S1. Site Characterization

Complete metadata for all 18 sampling sites, including geographic coordinates, urban classification, exposure level, location descriptions, and surface materials, are provided in Table S1. Industrial-High (IH) sites were positioned on building facades within 50 m of identified emission sources in the Torch High-tech Zone (electronics manufacturing) and Haicang Industrial District (chemical processing). Industrial-Low (IL) sites were located on perimeter walls of the same industrial zones but >200 m from point sources. Traffic-High (TH) sites included BRT viaduct columns, tunnel entrance guardrails, and bridge piers along major traffic corridors (>50,000 vehicles day^{-1}). Traffic-Low (TL) sites were on building facades approximately 50 m leeward of major roads. Control-Campus (CC) sites were within vegetated areas of Xiamen University, >500 m from major roads or industrial facilities. Control-Gulangyu (CG) sites were on historic buildings in the UNESCO-listed Gulangyu Island, where motorized traffic is prohibited (UNESCO, 2017).

S2. Chemical Methods

S2.1 Auxiliary chemical parameters

Soil pH was measured in a 1:2.5 soil-water suspension using a calibrated glass electrode (Mettler Toledo). Amorphous iron and aluminum oxides (Fe-ox, Al-ox) were extracted with acid ammonium oxalate in darkness (Schwertmann, 1964) and quantified by ICP-OES (Thermo iCAP 7400). Total organic carbon (TOC) was determined by dry combustion using a TOC analyzer (Elementar vario TOC cube) after removal of inorganic carbonates with HCl. These parameters are included in the seasonal datasets (Tables S2, S3).

S2.2 Quality assurance and quality control

For all chemical analyses, method detection limits (MDLs) were: As (0.05 mg kg^{-1}), P (5 mg kg^{-1}), Fe-ox (0.1 g kg^{-1}), Al-ox (0.1 g kg^{-1}), and TOC (0.5 g kg^{-1}). Certified

reference materials (CRMs) analyzed with each batch included GBW07405 (soil) for solid samples and SLRS-6 (river water) for runoff samples. Spike recoveries ranged from 92–108%. Duplicate analyses of 10% of samples showed relative percent differences <10% for all analytes.

S3. Molecular Methods

S3.1 qPCR primer information

Primer sequences and thermal cycling conditions for all target genes are provided in Table S4. The *arsC* gene was amplified using primers amlt-42-f/amlt-376-r (). The *arsM* gene was targeted with primers arsM-F/arsM-R (Jia *et al.*, 2013). Phosphorus cycling genes *pstS* and *phoD* were quantified using primers pstS-F/pstS-R (Lidbury *et al.*, 2016) and ALPS-F730/ALPS-R1101 (Sakurai *et al.*, 2008), respectively. For greenhouse gas-related genes, *mcrA* was amplified with mlas-F/mcrA-R (Steinberg and Regan, 2008), and *dsrB* with DSRp2060F/DSR4R (Wagner *et al.*, 1998). Each 20-µL qPCR reaction contained 10 µL SYBR Green Master Mix (Applied Biosystems), 0.4 µM each primer, and 2 µL template DNA (1–10 ng).

S3.2 Sequencing data processing

Raw sequence reads were demultiplexed based on unique barcode combinations. DADA2 processing included quality filtering (maxEE = 2, truncQ = 2), dereplication, error model learning, and chimera removal using the consensus method (Callahan *et al.*, 2016). Amplicon sequence variants (ASVs) with <0.1% relative abundance across all samples were removed to reduce spurious diversity. After quality filtering, an average of 45,000 reads per sample were retained (range: 32,000–62,000). Rarefaction curves indicated adequate sequencing depth for all samples (data not shown). Beta diversity patterns were assessed using Bray-Curtis dissimilarity and visualized with non-metric multidimensional scaling (NMDS), confirming clear separation of microbial communities by interface type (Figure S3).

S4. Microcosm Incubation Details

Anaerobic microcosms were established in triplicate for each of the 18 sampling sites. The mineral medium contained (per liter): NaCl (1.0 g), $MgCl_2 \cdot 6H_2O$ (0.4 g), $CaCl_2 \cdot 2H_2O$ (0.1 g), NH_4Cl (0.25 g), KH_2PO_4 (0.2 g), and trace elements (Wolin *et al.*, 1963). No exogenous carbon source was added to assess endogenous organic matter mineralization. Headspace (100 mL) was flushed with O_2-free N_2 for 5 min prior to sealing. Bottles were incubated horizontally with gentle shaking (50 rpm). Gas samples were analyzed immediately after collection using a GC equipped with a Porapak Q column (80/100 mesh) at 80°C, with He as carrier gas. CH_4 and CO_2 were detected by FID (with methanizer) and TCD, respectively. Detection limits were 0.5 ppm for CH_4 and 50 ppm for CO_2. Fluxes were calculated assuming ideal gas behavior and normalized to the surface area of sampled interfaces.

Table S1. Sampling site metadata for 18 urban interface locations in Xiamen, China.

Sample ID	Interface type	Urban type	Exposure	Location description	Surface material	Height (m)
IH-1	Industrial-High	Industrial	High	Torch High-tech Zone, facade near emission sources	Concrete/coated	1.3
IH-2	Industrial-High	Industrial	High	Haicang Industrial District, facade near emission	Concrete/coated	1.3
IH-3	Industrial-High	Industrial	High	Torch High-tech Zone, facade near emission sources	Concrete/coated	1.3
IL-1	Industrial-Low	Industrial	Low	Industrial zone perimeter wall, background site	Concrete wall	1.3
IL-2	Industrial-Low	Industrial	Low	Industrial zone perimeter wall, background site	Concrete wall	1.3
IL-3	Industrial-Low	Industrial	Low	Industrial zone perimeter wall, background site	Concrete wall	1.3
TH-1	Traffic-High	Traffic	High	BRT viaduct columns, high traffic exposure	Concrete/metal	1.3
TH-2	Traffic-High	Traffic	High	Tunnel entrance guardrails	Concrete/metal	1.3
TH-3	Traffic-High	Traffic	High	Bridge piers, high traffic exposure	Concrete/metal	1.3
TL-1	Traffic-Low	Traffic	Low	Buildings ~50 m from viaduct, leeward	Building facade	1.3
TL-2	Traffic-Low	Traffic	Low	Noise barrier leeward side	Building facade	1.3
TL-3	Traffic-Low	Traffic	Low	Buildings ~50 m from viaduct, leeward	Building facade	1.3
CC-1	Control-Campus	Control	Low	Xiamen University of Technology campus, green area	Building facade	1.3
CC-2	Control-Campus	Control	Low	Xiamen University of Technology campus, green area	Building facade	1.3
CC-3	Control-Campus	Control	Low	Xiamen University of Technology campus, green area	Building facade	1.3
CG-1	Control-Gulangyu	Control	Low	Gulangyu Island, heritage conservation zone	Historic building	1.3
CG-2	Control-Gulangyu	Control	Low	Gulangyu Island, heritage conservation zone	Historic building	1.3
CG-3	Control-Gulangyu	Control	Low	Gulangyu Island, heritage conservation zone	Historic building	1.3

Note: All samples collected from vertical urban interfaces at 1.3 m height. Sampling period: dry season (Oct-Dec 2024) and wet season (May-Jul 2024).

Table S2. Seasonal variation in arsenic transformation gene abundances.

Interface type	*arsC* (Dry)	*arsC* (Wet)	*arsM* (Dry)	*arsM* (Wet)	*pstS* (Dry)	*pstS* (Wet)
Industrial-High	7.34 ± 0.25	7.43 ± 0.07	6.90 ± 0.15	6.65 ± 0.09	7.13 ± 0.24	7.17 ± 0.14
Industrial-Low	6.83 ± 0.43	6.79 ± 0.21	6.42 ± 0.34	6.33 ± 0.06	6.92 ± 0.21	7.12 ± 0.03
Traffic-High	6.41 ± 0.13	6.42 ± 0.04	6.01 ± 0.18	6.13 ± 0.15	6.65 ± 0.02	6.82 ± 0.27
Traffic-Low	6.05 ± 0.13	6.14 ± 0.10	5.60 ± 0.16	5.82 ± 0.12	6.49 ± 0.13	6.43 ± 0.06
Control-Campus	5.64 ± 0.19	5.79 ± 0.15	5.28 ± 0.34	5.47 ± 0.24	6.14 ± 0.22	6.35 ± 0.18
Control-Gulangyu	5.76 ± 0.11	5.83 ± 0.08	5.55 ± 0.14	5.62 ± 0.11	6.12 ± 0.08	6.05 ± 0.05

Note: Values are mean ± SD (n = 3). Gene abundances expressed as $\log_{10}$ copies g^{-1} dry weight. Dry season: Oct-Dec 2024; Wet season: May-Jul 2024.

Table S3. Pearson correlation coefficients among chemical and microbial parameters.

	**As**	**P**	**As/P**	**OlsenP**	***arsC***	***arsM***	***pstS***	***phoD***	**Shannon**
As	1.00								
P	-0.76***	1.00							
As/P	0.97***	-0.76***	1.00						
OlsenP	-0.78***	0.72***	-0.72***	1.00					
arsC	0.87***	-0.86***	0.82***	-0.84***	1.00				
arsM	0.89***	-0.86***	0.85***	-0.79***	0.95***	1.00			
pstS	0.76***	-0.81***	0.73***	-0.78***	0.88***	0.80***	1.00		
phoD	-0.67**	0.52*	-0.64**	0.66**	-0.61**	-0.62**	-0.46	1.00	
Shannon	-0.84***	0.71**	-0.86***	0.69**	-0.76***	-0.74***	-0.77***	0.59**	1.00

Note: *p < 0.05, **p < 0.01, ***p < 0.001. n = 18 samples. Lower triangle shows Pearson r values.

Table S4. Primers used for quantitative PCR of functional genes

Target gene	Function	Primer sequence (5′→3′)	Size (bp)	Reference
arsC	Arsenate reductase	F: GGTGTGGAACATCGTCTGGA R: CAGGCCGTACACCACCGGCT	334	
arsM	Arsenic methyltransferase	F: TCYCTCGGCTGCGGCAAYCCVAC R: CGWCCGCCWGGCTTWAGYACCCG	348	Jia et al., 2013
pstS	Phosphate transporter	F: ATGATYACVATGAARGAYGG R: CCANCCYTTRTGDATRTTYTC	287	Lidbury et al., 2016
phoD	Alkaline phosphatase	F: CAGTGGGACGACCACGAGGT R: GAGGCCGATCGGCATGTCG	371	Sakurai et al., 2008
mcrA	Methyl-CoM reductase	F: GGTGGTGTMGGDTTCACMCARTA R: CGTTCATBGCGTAGTTVGGRTAGT	470	Steinberg and Regan, 2008
dsrB	Dissimilatory sulfite reductase	F: CAACATCGTYCAYACCCAGGG R: GTGTAGCAGTTACCGCA	361	Wagner et al., 1998

Note: Degenerate bases: Y = C/T; W = A/T; R = A/G; M = A/C; D = A/G/T; V = A/C/G; B = C/G/T. All reactions performed with SYBR Green chemistry on QuantStudio 6 Flex (Thermo Fisher). qPCR conditions: 95°C 10 min; 40 cycles of 95°C 15 s, Tm 30 s, 72°C 30 s.

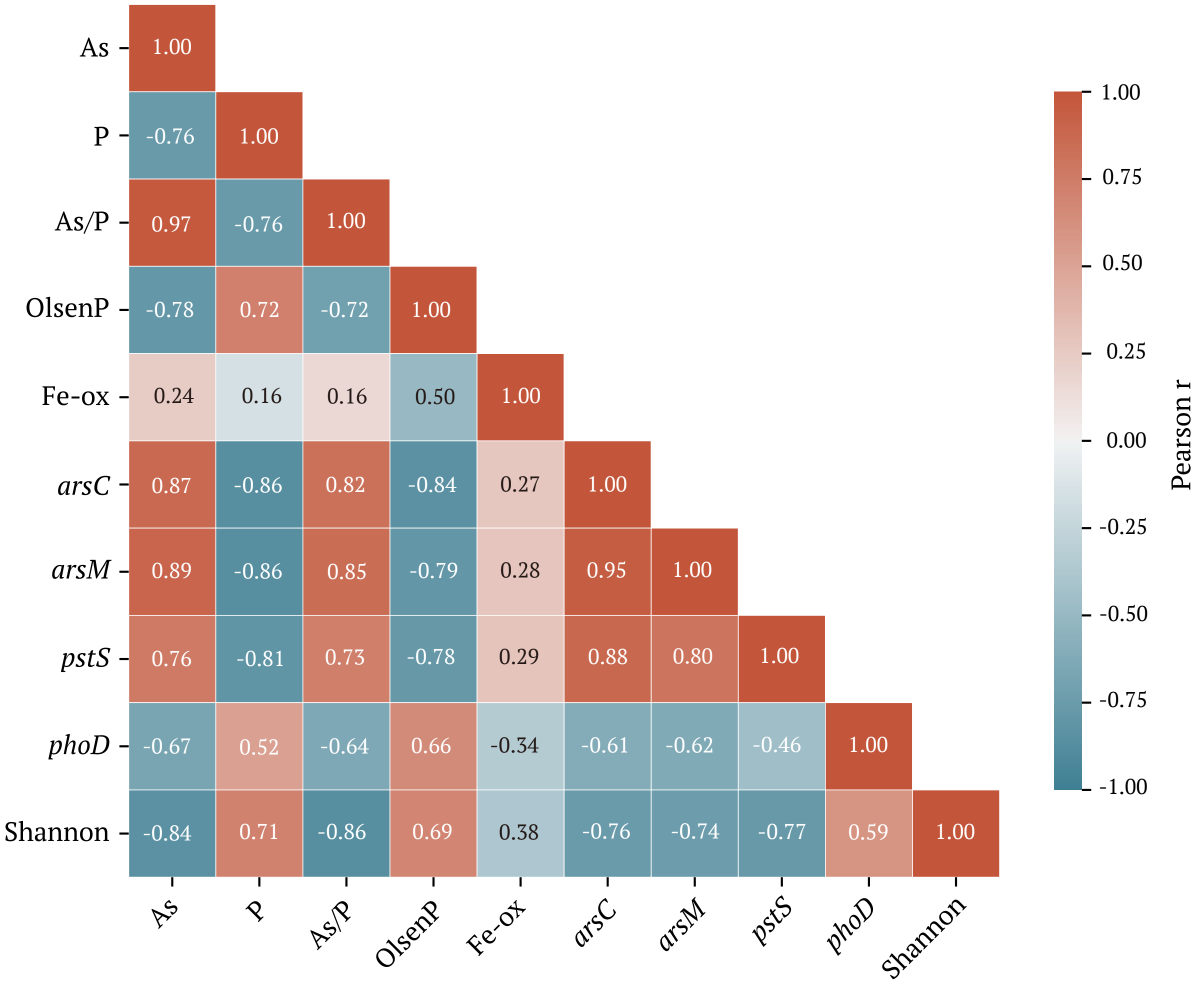

Figure S1. Correlation heatmap of chemical and microbial indicators. Lower-triangle Pearson correlation matrix for all measured variables (n = 18). The dashed boxes highlight two major parameter clusters: chemical indicators (upper left, bounded by red dashed line) and microbial functional genes (lower right, bounded by blue dashed line). Color scale indicates correlation strength: red = positive correlation; blue = negative correlation. Key findings include: (1) strong negative correlation between As and P ($r = -0.76$, $p < 0.001$); (2) strong positive correlations among As-related genes (*arsC*, *arsM*, *pstS*; $r > 0.80$); (3) *phoD* shows inverse pattern to other functional genes. Exact correlation coefficients and significance levels are provided in **Table S3**.

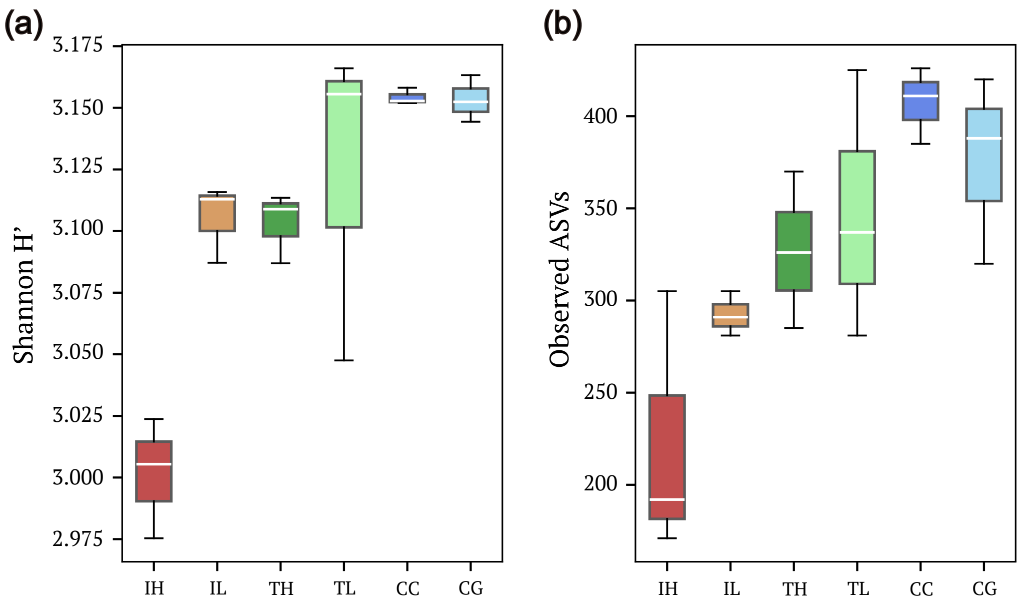

Figure S2. Alpha diversity of microbial communities across urban interface types. (A) Shannon diversity index (H') showing reduced diversity at high-exposure industrial sites (IH: 3.00 ± 0.02) compared to control sites (CC: 3.15 ± 0.00; CG: 3.15 ± 0.01). (B) Observed amplicon sequence variants (ASVs) demonstrating similar pattern of diversity reduction under high As stress. The negative correlation between Shannon diversity and As concentration ($r = -0.84$, $p < 0.001$; **Table S3**) suggests that arsenic contamination exerts selective pressure on microbial communities, favoring As-tolerant taxa while reducing overall diversity. Box plots show median (horizontal line), interquartile range (box), and range (whiskers).

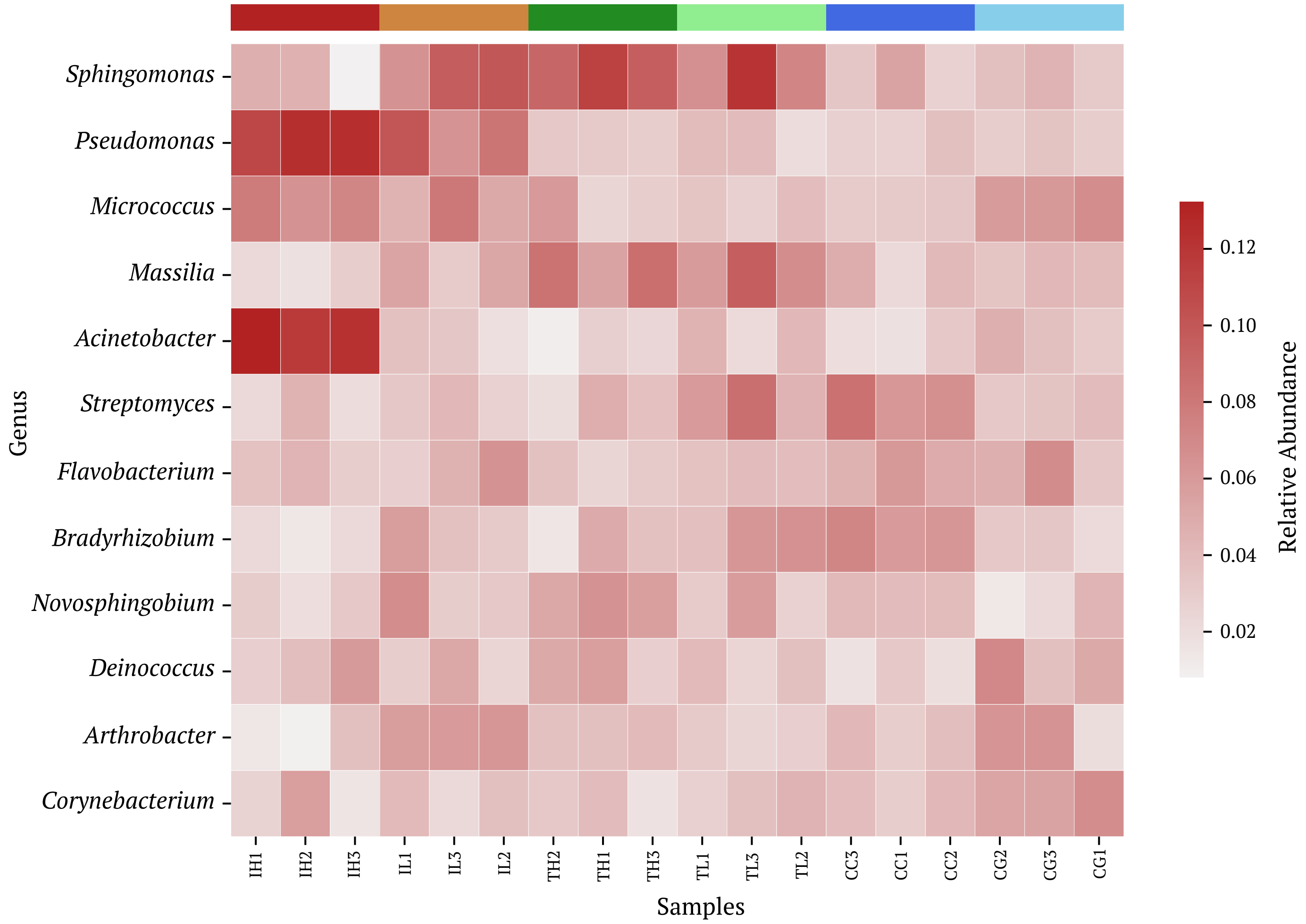

Figure S3. Bacterial community composition at genus level across urban interface samples. Heatmap showing relative abundance of the top 12 bacterial genera across 18 samples. Samples are ordered by group (indicated by color bar at top). Notable patterns include: (1) Acinetobacter and Pseudomonas show enrichment at industrial sites (IH), consistent with their known arsenic resistance capabilities; (2) *Sphingomonas* and *Methylobacterium* are more abundant at control sites (CC, CG), indicating preference for lower-stress environments. These community shifts correspond to the functional gene patterns observed in **Figure 2A** and support the selective enrichment of As-tolerant microorganisms at contaminated interfaces.

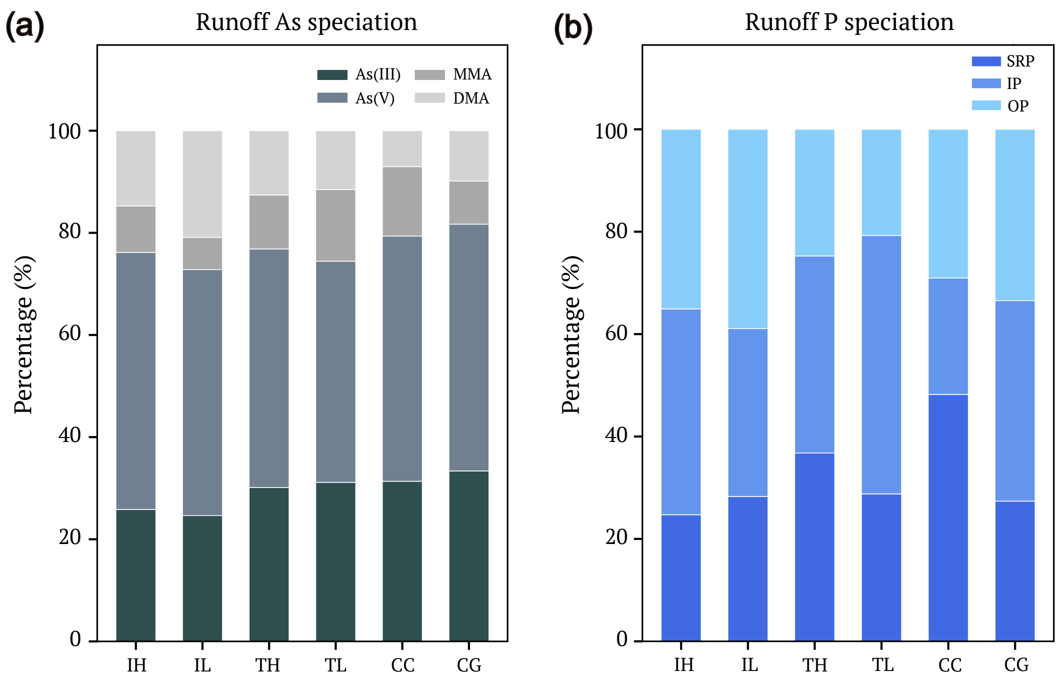

Figure S4. Detailed speciation of arsenic and phosphorus in stormwater runoff. (A) Arsenic speciation in runoff showing proportions of arsenite [As(III)], arsenate [As(V)], monomethylarsonic acid (MMA), and dimethylarsinic acid (DMA). The presence of methylated species (MMA + DMA, comprising 18–27% across groups; **Table 2**) provides direct evidence of microbial *arsM*-mediated transformation. (B) Phosphorus fractions in runoff showing soluble reactive P (SRP), inorganic P (IP), and organic P (OP) proportions. Higher SRP percentage at control sites (CC: 48%) compared to industrial sites (IH: 25%) indicates greater P bioavailability in low-As environments. These speciation data complement the summary statistics in **Figure 3** and **Table 2**.

Reference

Callahan, B. J., McMurdie, P. J., Rosen, M. J., Han, A. W., Johnson, A. J. A., and Holmes, S. P. (2016). DADA2: High-resolution sample inference from Illumina amplicon data. *Nat. Methods* 13, 581–583. doi: 10.1038/nmeth.3869

Jia, Y., Huang, H., Zhong, M., Wang, F. H., Zhang, L. M., and Zhu, Y. G. (2013). Microbial arsenic methylation in soil and rice rhizosphere. *Environ. Sci. Technol.* 47, 3141–3148. doi: 10.1021/es303649v

Lidbury, I. D. E. A., Murphy, A. R. J., Sherlock, D. J., Sherlock, A., and Chen, Y. (2016). Comparative genomic, proteomic and exoproteomic analyses of three *Pseudomonas* strains reveals novel insights into the phosphorus scavenging capabilities of soil bacteria. *Environ. Microbiol.* 18, 3535–3549. doi: 10.1111/1462-2920.13390

McMurdie, P. J., and Holmes, S. (2013). phyloseq: An R package for reproducible interactive analysis and graphics of microbiome census data. *PLoS ONE* 8, e61217. doi: 10.1371/journal.pone.0061217

Sakurai, M., Wasaki, J., Tomizawa, Y., Shinano, T., and Osaki, M. (2008). Analysis of bacterial communities on alkaline phosphatase genes in soil supplied with organic matter. *Soil Sci. Plant Nutr.* 54, 62–71. doi: 10.1111/j.1747-0765.2007.00210.x

Schwertmann, U. (1964). Differenzierung der Eisenoxide des Bodens durch Extraktion mit Ammoniumoxalat-Lösung. *Z. Pflanzenernähr. Düng. Bodenk.* 105, 194–202. doi: 10.1002/jpln.3591050303

UNESCO, W. (2017). Kulangsu, a historic international settlement.

Steinberg, L. M., and Regan, J. M. (2008). Phylogenetic comparison of the methanogenic communities from an acidic, oligotrophic fen and an anaerobic digester treating municipal wastewater sludge. *Appl. Environ. Microbiol.* 74, 6663–6671. doi: 10.1128/AEM.00553-08

Wagner, M., Roger, A. J., Flax, J. L., Brusseau, G. A., and Stahl, D. A. (1998). Phylogeny of dissimilatory sulfite reductases supports an early origin of sulfate

respiration. *J. Bacteriol.* 180, 2975–2982. doi: 10.1128/JB.180.11.2975-2982.1998

Wolin, E. A., Wolin, M. J., and Wolfe, R. S. (1963). Formation of methane by bacterial extracts. *J. Biol. Chem.* 238, 2882–2886. doi: 10.1016/S0021-9258(18)67912-8
